# “Ultimately, mom has the call”: Viewing clinical trial decision making among patients with ovarian cancer through the lens of relational autonomy

**DOI:** 10.1111/hex.12691

**Published:** 2018-04-14

**Authors:** Gladys B. Asiedu, Jennifer L. Ridgeway, Katherine Carroll, Aminah Jatoi, Carmen Radecki Breitkopf

**Affiliations:** ^1^ Robert D. and Patricia E. Kern Center for the Science of Health Care Delivery Mayo Clinic Rochester MN USA; ^2^ Department of Health Sciences Research Mayo Clinic Rochester MN USA; ^3^ College of Arts and Social Sciences Australian National University Canberra ACT Australia; ^4^ Department of Oncology Mayo Clinic Rochester MN USA

**Keywords:** clinical trial, family members, oncology, ovarian cancer, qualitative, relational autonomy, trial decision making

## Abstract

**Objective:**

This study employs the concept of relational autonomy to understand how relational encounters with family members (FMs) and care providers may shape decisions around ovarian cancer patients’ clinical trial (CT) participation. The study also offers unique insights into how FMs view patients’ decision making.

**Methods:**

In‐depth interviews were conducted with 33 patients with ovarian cancer who had been offered a CT and 39 FMs. Data were inductively analysed using a thematic approach and deductively informed by constructs derived from the theory of relational autonomy (RA).

**Results:**

Patients’ relationships, experiences and social status were significant resources that shaped their decisions. Patients did not give equal weight to all relationships and created boundaries around whom to include in decision making. Doctors’ recommendations and perceived enthusiasm were described as influential in CT decisions. Both patients with ovarian cancer and their FMs maintained that patients have the “final say,” indicating an individualistic autonomy. However, maintaining the “final say” in the decision‐making process is constitutive of patients’ relationships, emphasizing a relational approach to autonomy. FMs support patients’ autonomy and they do so particularly when they believe the patient is capable of making the right choices.

**Conclusions:**

Although ethical principles underlying informed consent for CT participation emphasize individual autonomy, greater attention to relational autonomy is warranted for a more comprehensive understanding of CT decision making.

## 1 INTRODUCTION

Potential research participants in clinical trials (CTs) must be informed of their right to self‐determination and be provided an in‐depth description of foreseeable benefits, risks of the trial and alternatives to participation as part of the process of informed consent.^1^ Rational decision models and shared decision‐making approaches focus on the individual patient's autonomy in CT decision making.^2^, ^3^, ^4^, ^5^ However, social circumstances, including experiences, education, relationships and racial and cultural identification, all of which inform one's selfhood and therefore one's decision making, may result in departures from idealized, standardized or anticipated decision processes.^6^ Indeed, reports indicate that the process by which patients with cancer make CT participation decisions is diverse and poorly understood.^6^, ^7^ The decision‐making process itself can be viewed as a “silent factor” in CT decision making, although much of the literature assumes shared decision making among patients, providers and family members (FMs) is preferred.^6^

Research on ethical CT presentation highlights the need to enhance patients’ autonomy by meeting ethical obligations and reforming informed consent processes so that they are an ongoing interactive process that prioritizes patients’ privacy as well as creates opportunities for patients to interact with their social network.^2^, ^4^, ^5^, ^8^, ^9^ Understanding how patient's autonomy is enacted is significant to the broader CT participation literature as it can inform efforts that seek to optimize trial enrolment among patients with cancer. However, there has been little focus on how patients’ autonomy is embedded within family involvement in treatment decision making.^10^, ^11^, ^12^, ^13^ Illness is not an isolated event that occurs at the individual level, but rather it can be a challenging circumstance that evolves from a family's history, and which can impact its future.

The objective of this research was to understand the social and familial contexts that shape cancer patients’ and their family members’ decision to enrol in a CT focusing on how family and other social engagement promote patients’ overall agency. Specifically, we set out to understand patients’ perceptions of their experiences around who, when and how others are engaged in CT decision making, and how those interactions shape their decisions, as well as how FMs perceive their own participation. As a guiding concept, we employ the relational autonomy (RA) theory^14^ to understand these relational and social engagements. Relational autonomy is an umbrella term for a feminist reconfiguration of traditional notions of autonomy which are based upon a fixed, unchanging, independent and rationalistic conceptualization of an individual selfhood.^14^, ^15^

Within traditional notions of autonomy, informed consent and clinical decision making are viewed as an individual and rational exercise free from the influence of others.^2^, ^3^, ^4^ Instead, RA casts the individual's selfhood as iteratively shaped by, experienced and produced through interconnected relationships with others, and through the individual's own biography, emotions and social experiences.^14^, ^15^ Personal autonomy, therefore, is realized through a dynamic web of social, historical, class, racial, gendered and cultural contexts,^14^, ^15^, ^16^, ^17^ with inherent uneven power relations embedded within this complexus. In this article, we conceive RA as an approach that involves examining how one's sense of self, one's autonomy and one's decisions are developed and (re)confirmed in the context of daily interactions and experiences^14^, ^16^, ^18^ From the perspective of RA, clinical decision making and informed consent are considered to be shaped by, and dependent upon relationships with others^14^, ^15^, ^16^, ^17^ and deliberately, one's autonomy.

Ovarian cancer provides a highly relevant disease context in which to examine CT decision making. Rates of recurrence after treatment are high,^19^ and a majority of patients diagnosed with this cancer eventually face drug‐resistant disease and recurrence which limit treatment options to novel agents or regimens that are only available through CTs.^19^, ^20^, ^21^, ^22^ Social and disease factors including finances, family obligations and quality of life are important considerations in ovarian cancer treatment decision making.^23^ This presents an opportunity to explore the topic of autonomy in decision making—which is portrayed as rational model—in the context of a lethal disease where patients are faced with decisions that impact both their medical and social situation.

## 2 METHOD

### 2.1 Research approach

This study used an applied qualitative health research approach^24^ focusing on patients’ and FMs’ perspectives on their experiences around decision making in CT participation and how findings could be applicable in CT enrolment decisions. We used an integrated approach of applied thematic analysis^25^, ^26^ which involves a combination of inductive (themes emerging from participant's responses) and deductive (constructs from RA) coding. All participants provided informed consent and were assured of anonymity and confidentiality. Ethics approval was obtained prior to initiating the study.

### 2.2 Recruitment and data collection

The data for this study were collected from January 2012 through December 2014. Specific procedures surrounding methods and recruitment have been previously reported.^27^, ^28^ We used a purposeful sampling approach.^29^ Eligible patients had a diagnosis of ovarian cancer (epithelial ovarian carcinoma, primary peritoneal carcinoma or fallopian tube cancer), had been offered a CT (at one of two comprehensive cancer centres in the Midwest of the USA) and were willing to nominate at least one FM to participate in a separate interview. Nominated FMs were contacted to participate in an interview subsequent to enrolling the patient. Family members included biological and social relatives.

Data were gathered using a semi‐structured interview guide. Selected topics explored in the interviews included: perceptions around factors important to CT decision making; patients’ communications with FMs and the healthcare team; how and when FMs are involved in the decision‐making process; FM's role and influence; and how patients made final decisions on trial enrolment. Interview guides for patients and FMs were reviewed by the Mayo Clinic ovarian cancer patient advocate group. Interviews were conducted in‐person or by telephone. Each interview lasted approximately an hour and was audio recorded. Participants received a hand‐written card signed by their interviewer and an ovarian cancer awareness pin as an expression of gratitude for their time and participation.

### 2.3 Analysis

In an integrated approach, we began with an inductive analysis^25^, ^26^, ^30^ to identify major themes. Inductive data analysis was conducted by a team of five qualitative researchers in the areas of family studies, health services research, behavioural science, and medical sociology. Researchers met regularly to discuss and compare emerging themes, and categories. Subsequently, constructs from RA were applied as a sensitizing concept to the emergent themes, thereby allowing the analysis to be a process in which RA could explain the contexts within which patients with ovarian cancer make CT decisions, including their social context. Data management and analysis were aided by qualitative analysis software (NVIVO 11.1; QSR International PTY ltd.). Illustrative quotes are denoted by participant number; for patients, age (in years) follows, while for FMs, their gender and relationship to the patient follows their participant number.

## 3 RESULTS

Participant characteristics have been reported earlier.^27^, ^28^ Briefly, a total of 72 participants (33 patients with ovarian cancer and 39 FMs) were interviewed across both study sites. Patients’ mean age was 59 ± 9.9 (range 36‐76). Seventy percent (n = 23) of patients were married or in a relationship, and 30% were never married, widowed, separated or divorced (n = 10). More than half (58%) were not working for pay (n = 19), and 55% had a bachelor's degree or higher (n = 18). The vast majority of the patient sample (94%, n = 31) self‐identified their race/ethnicity as White, non‐Hispanic.

The majority (59% n = 23) of FMs was female. FMs identified as spouses/partners (33%, n = 13), adult children (23%, n = 9), siblings (15%, n = 6), parents (8%, n = 3), friends (15%, n = 6), niece (<1%, n = 1) and daughter‐in‐law (<1%, n = 1). FMs’ mean age was 56.0 ± 13.4 (range 25‐81); 59 percent had a bachelor's degree or higher (n = 23). More than half (54%, n = 21) were working full time; 22 percent (n = 28) were married or in a relationship, and 28% were never married, widowed, separated or divorced (n = 9). All FMs self‐identified their race/ethnicity as White, non‐Hispanic.

Findings for this study are categorized into three main themes that portray the relational aspects influential in patients’ decisions around CT participation. The first theme, “relational engagement in CT decisions among patients with ovarian cancer,” emphasizes the multiple relationships that influence patient's experience of selfhood and their decisions regarding CT participation. The second theme, “familial roles and perceptions of the patient's decisions,” is an account of how FMs perceive their role in the decision‐making process. Ultimately, patients make decisions regarding whether or not to participate in a CT that are informed by multiple relationships, which is discussed in the third theme “maintaining the final say.”

### 3.1 Relational engagement in CT decisions among patients with ovarian cancer

As Sherwin^14^ notes, analysis that emphasizes the standard conception of autonomy as described in bioethics discourages attention to the context in which decisions are actually made and obscures the need to question the influence of power or structural aspects of social class and social relationships in the interpretation of health‐related matters. Responses from patients in this study suggest that patients with cancer, as individuals, are socially constructed and the decisions they make around CT enrolment are constituted through their interactions with their social environment.

Revealing that patients’ decision making is not independent of their interactions, patients in this study described their motivation to acquire knowledge and support through family, friends and physicians before making decisions about CT participation. Not all relationships were considered equal, and differing levels of relational engagement and influence were described. Moreover, patients perceived that some interactions may enhance or undermine their decisions. To manage this, patients selectively involved FMs in their decision making, including determining when to share information and how to share particular types of information. Patients engaged FMs in decision making when they shared a strong and close relationship (especially with spouses and some adult children), or when FMs had scientific or medical training. Patients also sought experiential knowledge from other patients with cancer, enquiring about “what they tried and what they used” to help understand the reality of trial participation, including what side‐effects other patients had experienced.

Medical background or scientific expertise was a noted characteristic of FM inclusion in the decision‐making process, particularly those who could interpret CT information and discuss treatment options. Yet the same characteristic was also noted as potentially excluding FMs from the decision‐making process in some cases. For example, one patient recounted that she did not involve her daughter in decision making because her daughter's background in pharmacology had previously led her to make comments about CT participants being “guinea pigs.” Another patient [17a, age 68] recounted, *“…if I talk to somebody, well, they might hedge a little bit and then it makes you start thinking – are you making the right thing [decision]?”* Other reasons for excluding FMs from discussions about trial participation included a desire to avoid creating perceived emotional and psychological burdens for FMs, particularly when taking into consideration the age and health condition of FMs.

For many patients, the decision to enrol in a CT was based on knowledge acquired from physicians, and in some cases, information about the trial was presented as a recommendation to participate. Doctors’ comments that were reported by patients in the interviews such as, “you would be a good candidate for it,” “there is a silver lining in here” and “it's a great idea” were identified by patients as powerful in shaping their CT participation decisions. Given the complexity of CT information and treatment decisions in general, a doctor's recommendation or information obtained from internet searches had the power to shape how patients understood the relative benefits and risks of participating in a way that FMs did not. As such, a doctor's recommendation was seldom questioned by patients; although decisions were left to patients, patient understanding of the trial—and particularly, the trial's benefits—was shaped by physician language. “*He* [oncologist] *just told me that is what he thought I should do, so that is what I did – I just took his word for it. He said this is looking promising, you know, I think you should participate in this. So I never really did any research on my own – I just trusted him…I don't think I questioned to be in it because he suggested it. No, I think at that time we were just so excited that* [oncologist] *was excited.”* [10a, age 54] Where such recommendations were absent, some patients reported that they wished their doctors would have recommended a trial or told them what to do. Some providers reportedly promoted a particular trial as having had good results or suggested that a particular patient's participation was an opportunity to help not only the patient but also others as well. Some doctors reportedly told patients that specific trials being offered were geared towards patients’ needs “*And he said you are basically, a poster child for this because he said you are a perfect candidate*.” [27a, age 57].

RA asserts that one's educational, occupational and personal biographical experiences and characteristics inform the decisions one makes.^17^ For some patients, these personal characteristics and social positions were considered assets and resources that provided them with the necessary skills for making CT participation decisions. For example, patients perceived that being “independent,” having strengths in sound decision making or having a medical background were instrumental in their CT enrolment decisions. Patients without medical expertise mentioned other life‐experiences that helped them consider their options.

I have taken some biochemistry classes and some molecular biology classes… so I'm not as wary as some other people might be…– be as familiar with some of those processes, possibly ‐ or being interested in reading some of the journal articles.

[15a, age 36]

… in fact, because this is the nature of my former employment [school superintendent] before I was retired, I made a table of each [CT offered] and provided information so that I could compare the three.

[103a, age 64]

…everybody knows I make sound decisions at work, at home, family… I'm very analytical thinking and I just put things in perspective.

[24a, age 42]

Patients also considered the impact that CT participation would have on family circumstances or obligations. Travelling to the clinic for trial participation included overnight stays and transportation costs. It also meant being unavailable to perform family duties.

We live on a small farm, so the hassle – and we have a 15‐year‐old son, so our challenge is, well [son] being the priority, you know, be least disruptive to his life and then who is going to take care of the animals?

[1a, age 58]

I wouldn't want to put my family in financial ruin over…I'm sorry, I mean and maybe die anyway, I certainly wouldn't do that.

[105a, age 55]

For one patient, her participation in a trial meant overnight hospital stays. During that time, her mother stayed with her, and she talked about weighing the impact of trial participation on her mother when she was considering the trial.

I mean the commitment just kind of affects, my mother has a husband who has some medical problems at home, so we have to leave him at home. I mean, he does okay on his own, but it is something to consider and obviously she has to give up work.

[25a, age 46]

The RA theory, which highlights the social context of autonomous decision making, assisted in uncovering the variety of factors that patients perceived as having significant influence on the CT participation decision‐making process. As patients with cancer face the dilemma of CT participation decision making, it is clear that they do not isolate their life‐experiences, educational backgrounds, personal characteristics and interactions with their doctors and FMs. Rather, patients actively draw on these experiences, interactions, relationships, family roles and responsibilities as part of their CT enrolment decision making.

### 3.2 Familial roles and perceptions of the patient's decision

This theme considers how FMs perceive their role in the CT participation decision process and how they view patients’ autonomous decision making. Some FMs positioned themselves as passive while others saw themselves as active in the decision‐making process. When asked about how much control they had over the final decision, most of them said they had none, although they noted that they were comfortable with the decision made by the patient.

FMs in our study largely confirmed that the ultimate decision rests with the patient, especially in the light of their realization that the patient is the one who will bear the burden of trial participation, including potential side‐effects. Family members reported considering factors such as travel and cost, but they also spoke about a desire to make any concessions that would benefit the patient. Some FMs talked about their passivity being a precaution against negative outcomes of the CT and the potential for regret. Other FMs reported that they were willing to provide their opinions but that the patient was independent enough to make a decision.

In her final decision to do the trial, I don't think I had any control at all.

[105c, female friend]

I stayed out of the decision… and I said this is your body, your life and your decision, not mine.

[25b, mother]

There was no decision on my part because she already said I'm going to do it… I don't feel I have the right to tell her ‘no’ if she wants to do it.

[24b, male spouse]

I'd like to be very careful, in what I say and don't say it too soon because it could be a decision that from me saying it, that then she would turn it down and then it would harm her or she would lose her life.

[11b, male spouse]

However, from a relational viewpoint on patient decision making, FMs attended hospital appointments and participated in CT participation discussions, showed concern, provided physical and emotional support, shared expert knowledge and thereby influenced the decisions that were made.

Family member views of the CT were often also shaped by physician presentation of them, and their trust in the physician's expertise. In some cases, FMs spoke about the provider being more knowledgeable in terms of the science, even if they (the FM) had some medical background.

Because I just felt, even, me being a nurse, if they would have…there are things that would have been said that I would have said, well, you know, what are you talking about, that doesn't make any sense to me. If there were things that would have put up red flags to me, I definitely would have said something. But they are the experts and I can't argue chemo with anyone because I don't know it. So when they came in with a trial and explained it, a couple of trials for me, it was like, these are the ones they felt were the best choice for her.

[4e, daughter in law]

He keeps his pulse on things a lot better than I do. He has an MD PH.D, which, you know, even though I'm old enough to be his father, I respect his intellect and his experience with ovarian cancer as well as his keeping up on things. And he has a network of his own.

[1b, male spouse]

FMs also reported that the trials were often presented in such a way that they felt their decision making was constrained by the reality of the disease progression and the limited available options for the patient. This perspective was described as curtailing any other factors weighing on their decision making.

I guess, you know, even if I don't get a warm fuzzy from [oncologist], I mean, I respected her from what I know about her and have heard about her and how long she has been in this working with people with ovarian cancer. And, you know, the few science things she threw out of her mouth all made sense to me…I mean, again, I felt like with this disease, you have to have some hope and this was really the only one that was offered, you know as a potential thing…Like between [oncologist] saying it and just the fact that it has a potential benefit, it seemed to me like it was a no‐brainer.

[23b, female friend]

I think at that point, you have just been hit with a huge bullet, so when they said this is the best we can offer you, it is a great chemo, it is very…I mean they felt very…this is going to work. They used the term, like one in six can finish, so it is a very strong fighting chemo, why wouldn't you try it? Your life isn't in your hands anymore so, yup; you go with what they say. You do, you go with what they say at that point.

[28b, female adult child]

FMs’ opinions generally reflected absolute acceptance of the patient's choices. However, some FMs believed that the decision should be a family decision. These individuals would influence a shift in patients’ perspective or intervene if they felt the patient did not make the right decision.

But it has got to be a family decision! You know, the patient and the family and the doctors… she relies on us very heavily.

[104b, sister]

If she would have said no, I would have encouraged her to do it… and her husband and I think that we would have definitely convinced her had she chosen not to.

[5b, sister]

FMs were aware of how they might influence patients’ decisions and created boundaries around their engagements. While most FMs perceived patients’ decisions as being independent of them, their engagement in the decision‐making process emphasizes a relational approach to autonomy.

### 3.3 Maintaining the final say

Both patients with ovarian cancer and FMs maintained that patients have the final say on whether or not to participate in a CT. Some patients perceived that CT participation decisions were self‐generated: *“I didn't wait for someone to tell me… I kind of made this one on my own. I don't know if it is the right or the wrong decision, but I was kind of like in charge of it.”* [10a, age 54]. Some patients valued insights from relational encounters, but they did not seem to perceive this as inhibiting their autonomy in making the final decision. *“I mean I talk to everybody, but then I kind of do what I feel is right for me.”* [14a, age 67].

However, even “maintaining the final say” in the decision‐making process is actually constitutive of their social and structural characteristics and their relationships and interactions with family, friends and health‐care providers as powerfully portrayed in a daughter's comment, “*Ultimately, mom has the call, but she will look towards me*.” In fact, some patients stressed the importance of FMs in supporting their decisions and reported that decisions were made in partnership with FMs, as portrayed in the use of plural pronouns such as “we” and “our” in talking about the final decision. Joint decisions were attributed to pre‐existing family dynamics, that is, relationships and trust that lend itself to joint decision making. Few patients said they made their decision to participate because their relatives said they should.

Final decisions were also constrained by the health‐care system and the disease context itself. While each of the consent forms (and likely the discussion around them) explicitly stated that patients should take time to consider the trial and talk with family, friends and health‐care providers, in fact, several of the patients in this study described feeling pressure to make a decision. Some of this pressure was related to patients’ worry that taking time to consider a trial would only give the cancer time to advance, especially if they also described knowing the lethal progression of ovarian cancer: *“We don't want to go home and make up our mind the next day. We want to make up our mind up now and we want to get the next – you know, get the schedule going and make something happen.”* [13a, age 43] Some patients also described trial design factors or treatment decisions that precluded time for consideration:

So, not only that, but you had to get a physical beforehand if you would be even eligible. So that had to be done in a certain timeframe, and this study was starting and so I was a little behind. I needed to get it done ASAP in order to get in on the study. …and they were only allowing like… 5 people… And, so, it was basically, you know, oh, okay, you need to hurry because, if not, somebody else may get it or some other hospital may get it.

[102a, age 49]

… they had to…do an additional blood draw because that day that I saw [the gynecologic surgeon] they did all of my pre‐surgical workup. I had blood draw, an EKG, I had chest X‐ray, so they needed to know that day whether I wanted that extra blood draw done for the study.

[5a, age 62]

Unfortunately, because the clinical trial requires a port‐a‐cath and she was having surgery, the obvious time to do it was while she was having surgery.

[22b, male spouse]

Clinical trials were often presented alongside other treatment options, and the initial presentation for some patients was at the time when their provider was asking them to decide a course of care, for example, at the first appointment following a surgery. Furthermore, patients who travelled to the clinic from a greater distance described the need to make a decision while they were at the clinic because that decision would trigger a blood draw, medication order or test that needed to be completed before they left for home.

Yup, but, and then given the information then I had to decide and, um, I told them that I decided, you know, we decided right then and there because I didn't want to have to travel back down and go through more tests. So we decided.

[27a, age 57]

Finally, two participants in this study described negative experiences from taking information home for reflection and discussion with family. By the time those patients made a decision to participate, the trial slots were no longer available. These experiences thus shaped those patients’ subsequent views on the trial decision‐making process and the factors that influence autonomous decision making.

## 4 DISCUSSION

This study explored how social and familial contexts shape CT participation decisions among patients with ovarian cancer. Using RA as a guiding concept, we identify CT participation decision making as a complex relational process shaped by patients’ engagement with health‐care providers, FMs and friends, and informed by patients’ broader social history and contemporary social context. Our findings are suggestive that, rather than viewing autonomy as having independence from extraneous influences and simply having the capacity to act with intent,^31^ autonomy is both mediated and constituted by relational, familial, social, structural and situational dimensions and, thus, should be valued as such.^14^, ^15^, ^32^

In the health‐care domain, decisions are considered autonomous if patients are competent, have adequate information and understanding, and are free from explicit coercion.^2^, ^3^, ^4^, ^5^, ^14^ This understanding of autonomy limits the social conditions that structure patients’ selfhood, autonomy and subsequent decision making. A relational view of autonomy focuses not only on the particular decision being considered but also on how the decision relates to an individual's sense of self, and how they view themselves in the context of decision making. Patients in this study reflected on their personal philosophies, characteristics and life‐experiences as contributing to CT participation decision making. They also drew on the experiences of other patients with cancer to facilitate or justify their decisions to participate in a CT, as has been established elsewhere.^33^, ^34^, ^35^, ^36^

In a relational approach, autonomy is known to occur within and as a result of relationships and interactions^14^, ^15^, ^16^, ^36^ Within this web of interactions, there were varying levels of influence, and patients were selective as to who, how, and when to engage others. The aim of this study was to understand the views of patients and FMs, but congruent with other reports,^6^, ^37^, ^38^, ^39^ this study found that the role of the health‐care provider was critical. In fact, recommendations and endorsement from a health‐care provider were seen by some patients as more powerful than those provided by FMs and predominated in patient's decision‐making processes. Patients, particularly those with a devastating cancer, may position themselves as passive in decision making because they believe doctors are knowledgeable of their best interests and preferences. Such perceptions about providers and the inherent vulnerable nature of the patient in the patient–physician relationship can create a socially constrained situation for patients to make decisions to participate in CT. Previous work^40^ notes how physicians and health‐care systems play significant roles in patients' autonomous decision making. Similarly, patients in this study referenced how physician descriptions of CT and the manner in which trials are presented shaped their decision to participate. Furthermore, health‐care system and CT design factors were reported as constraints on enrolment decisions. Our findings support Sherwin's^14^ argument that patients may have limitations in making autonomous decisions because the options presented to them have already been constructed in ways that are limiting to their autonomy. While relying on a health‐care provider's expert suggestion can be considered a proactive choice, patients may be compelled to comply with their care providers, thereby overriding their own preferences as they see providers as expert advisers.^14^ Health‐care providers have been looked to as masters of medical knowledge and maintain a fair amount of social power in consultations with patients.^41^ This power has been central to analyses of decision making and may indicate that relational aspects of trust in providers are more important decision factors than CT risks and benefits. These findings pose an important question for health‐care professionals to consider how their presence and communication style may influence patients' decision‐making processes. Furthermore, our findings that FMs put their faith in providers and relegate their own opinions to be secondary to those of providers highlights the importance of strategies that value the multiple dimensions of relational‐situated decisions. The current focus on decision‐making dyads^37^, ^38^, ^39^, ^40^ could be expanded to appreciate the interplay among patients, FMs and providers.

There is little empirical evidence about FM involvement in medical decision making,^13^ and concerns about patient autonomy may arise if FM beliefs differ from those of patients.^42^ Yet even in times of disagreement with FMs, patients described relationally informed decision‐making behaviours. For example, patients conveyed appreciation for the support of FMs in their decision‐making process, but would selectively involve certain FMs in CT participation decisions or avoid involving FMs whom they perceived might offer a contrary opinion.

These findings suggest that patients generally hold a relational perception of autonomy. Patients engage with relatives and friends, and they want their FMs to be involved in the decision‐making process. FMs, too, want to be involved in the decision‐making process, especially if their involvement conveys support of the patient. Both patients and FMs relegate the final decision to the patient, yet the final decision is a result of interactions within a social context, situational and structural context. Additionally, final CT enrolment decisions are constrained by health‐care systems and disease context. Some patients in this study reported being constrained by the confines of the disease and its poor prognosis, CT eligibility requirements, other treatment options and other factors that precluded time for consideration. The health‐care systems and CT eligibility requirements should consider how their processes influence autonomous decision making. Relational autonomy provides important theoretical perspectives to the CT participation decision‐making literature. In this view, the beliefs around a person's autonomy are expanded from a rationalistic discrete choice to an understanding of choice resulting from partnerships and engagements. Our findings echo calls for “person‐centred” approaches in shared decision making that emphasize patient experiences, relationships and interactions with their social world,^43^, ^44^ as well as models of decisions that emerge out of interaction.^40^

Decision‐making processes that view patients as the only decision makers may contribute to feelings of helplessness and isolation, cutting the patient off from others.^45^ Relationships with family are a part of patients' identity and context. It is therefore reasonable to assume that patients are concerned about how their decisions might affect FMs, and likewise, FMs are concerned about how patients' choices may affect their well‐being. Valuing patients and FMs in this way contributes to maintaining rather than inhibiting their relational autonomy and FMs as potential participants in decision‐making processes. Furthermore, patients in this study often talked about the importance of considering factors such as cost and travel, and how those might impact FMs, but the FMs in this study often framed considerations like these in the light of a desire to do anything possible to benefit the patient. Person‐centred approaches that consider the social context of patients' lives may further benefit from strategies to engage patients and FMs in difficult conversations such as these. This may be especially important in the context of ovarian and other gynaecological cancers where geographic access to specialty care and clinical trials is limited.^46^

There are limitations to this study. Although an effort was made to include patients with ovarian cancer and FMs from racial and ethnic minority populations, the majority of participants in this study identified themselves as non‐Hispanic White. Cultural factors have been reported to affect attitudes towards family involvement in the medical decision‐making process^47^ so these findings should not be generalized to diverse populations without further research. Also all patients were diagnosed with ovarian cancer which is known for its poor prognosis. Further evidence is required to examine whether cancer type influences FM roles, attitudes and perceptions surrounding CT participation decision making. Furthermore, future research should include health care providers’ perspectives on patients' CT decision making to understand how this coincides with patient and FM perspectives on health‐care provider influence. Despite these limitations, our study is significant; notably, the inclusion of the FM perspective serves to contextualize our assertion that efforts to understand CT participation decision making must be attentive to the importance of the relational context in which disease is experienced.

## 5 CONCLUSION

The findings of this study suggest that the concept of autonomy in health care in general, and CT participation decision making in particular, should consider the relational contexts, disease factors, health‐care system and CT design factors that shape patient decisions. Central to decision making is that patients reflect on their own and others’ views when they consider trial participation. Interactions between patients and their FMs as well as how patients interpret/understand health‐care providers’ recommendations for CT are important in patients’ decisional autonomy.

## CONFLICT OF INTERESTS

None.
